# Smad2 and Smad3 have differential sensitivity in relaying TGFβ signaling and inversely regulate early lineage specification

**DOI:** 10.1038/srep21602

**Published:** 2016-02-24

**Authors:** Ling Liu, Xu Liu, Xudong Ren, Yue Tian, Zhenyu Chen, Xiangjie Xu, Yanhua Du, Cizhong Jiang, Yujiang Fang, Zhongliang Liu, Beibei Fan, Quanbin Zhang, Guohua Jin, Xiao Yang, Xiaoqing Zhang

**Affiliations:** 1 Shanghai Tenth People’s Hospital, and Neuroregeneration Key Laboratory of Shanghai Universities, Tongji University School of Medicine, Shanghai 200092, China; 2 Department of Anatomy and Neurobiology, the Jiangsu Key Laboratory of Neuroregeneration, Nantong University, Jiangsu 226001, China; 3 The School of Life Sciences and Technology, Tongji University, Shanghai 200092; 4 Tongji University Advanced Institute of Translational Medicine, Shanghai 200092, China; 5 State Key Laboratory of Proteomics, Genetic Laboratory of Development and Diseases, Institute of Biotechnology, Beijing 100071, China; 6 The Collaborative Innovation Center for Brain Science, Tongji University, Shanghai 200092, China

## Abstract

The transforming growth factor beta (TGFβ) related signaling is one of the most important signaling pathways regulating early developmental events. Smad2 and Smad3 are structurally similar and it is mostly considered that they are equally important in mediating TGFβ signals. Here, we show that Smad3 is an insensitive TGFβ transducer as compared with Smad2. Smad3 preferentially localizes within the nucleus and is thus sequestered from membrane signaling. The ability of Smad3 in oligomerization with Smad4 upon agonist stimulation is also impaired given its unique linker region. Smad2 mediated TGFβ signaling plays a crucial role in epiblast development and patterning of three germ layers. However, signaling unrelated nuclear localized Smad3 is dispensable for TGFβ signaling-mediated epiblast specification, but important for early neural development, an event blocked by TGFβ/Smad2 signaling. Both Smad2 and Smad3 bind to the conserved Smads binding element (SBE), but they show nonoverlapped target gene binding specificity and differential transcriptional activity. We conclude that Smad2 and Smad3 possess differential sensitivities in relaying TGFβ signaling and have distinct roles in regulating early developmental events.

TGF-β superfamily agonists TGFβ, Activin, Nodal, bone morphogenetic proteins (BMPs) and growth and differentiation factors (GDFs) bind to TGFβ type II receptors and induce phosphorylation of TGFβ type I receptors^1^,^2^,^3^,^4^,^5^. Activated type I receptors phosphorylate regulatory Smads (R-Smads) at their C-terminal conserved Ser-Ser-X-Ser (SSXS) motifs^6^,^7^. These phosphorylated R-Smads oligomerize with common Smad (Co-Smad), Smad4, and then translocate into the nucleus, where they bind target gene promoters/enhancers and regulate gene transcription. In general, R-Smads which transduce BMP branch ligands (BMPs/GDFs) are Smad1/5/8, while Smad2/3 preferentially relay extracellular TGFβ branch signals (TGFβ/Activin/Nodal). These R-Smads are structurally similar as they are all characterized by two conserved regions known as the N-terminal Mad homology domain-1 (MH1) and C-terminal Mad homology domain-2 (MH2), which are separated by a linker region^4^,^8^.

Smad2 and Smad3 are closely related TGFβ downstream effectors with 92% amino acid sequence similarity^8^. To date, differences of Smad2 and Smad3 in signaling TGFβ ligands and their differential roles in chemistry and biology are still debating. It is mostly considered that Smad2 and Smad3 are equally important in mediating TGFβ signals and they are functionally interchangeable^7^,^9^,^10^,^11^,^12^,^13^. Upon TGFβ ligands stimulation, both Smad2 and Smad3 show SSXS phosphorylation, oligomerization with Smad4 and nuclear accumulation^7^,^11^,^12^. Knockout of Smad2 in mice results in early embryonic lethality due to failure to form primitive streak and defects in three germ layer patterning^14^,^15^,^16^. In chimera studies, Smad2 deficient embryonic stem cells fail to contribute to definitive endoderm^16^,^17^. These phenotypes are reminiscent with those observations in embryos with decreased levels of Nodal^17^. In contrast, Smad3 null mice are viable and fertile^18^,^19^,^20^. The dramatic phenotypic differences observed between Smad2 and Smad3 mutant mice may result from their uncompensatory expression pattern in the early embryo, since reconstitution of Flag-tagged Smad3 into the endogenous Smad2 locus partially rescues the lethality of Smad2 mutant mice, suggesting overlapped role of Smad2 and Smad3^13^.

At the biochemistry level, several lines of evidence demonstrate obvious differences between Smad2 and Smad3. In the basal state, Smad2 is found mostly as monomer, whereas Smad3 exists in multiple oligomeric states^21^. Smad3, but not Smad2, binds DNA through the β-hairpin DNA-interaction motif within the MH1 region^22^,^23^,^24^. It is also reported that TGFβ mediated Smad2/Smad4 or Smad3/Smad4 oligomers translocate into the nucleus through direct interaction with nuclear pore complex CAN/NUP214 and NUP153, while Smad3/Smad4 could also enter nucleus through an importin-β1 and Ran dependent mechanism^25^,^26^,^27^,^28^. However, whether Smad2 and Smad3 compensate, cooperate or antagonize with each other in order to finely shape TGFβ signals as well as downstream biological effects through these distinct biochemical characteristics remains to be resolved^11^,^29^,^30^,^31^.

In this study, we present evidence and show that in contrast to Smad2, which resides in the cytoplasm, a large pool of Smad3 preferentially distributes in the nucleus. More importantly, this static nuclear distribution of Smad3 is TGFβ signaling-, C-terminal phosphorylation- and Smad4-independent. Mutation studies verify that lack of the 30 amino acid sequence cognate to Smad2 exon 3 within the MH1 region is the key determinant for this noncanonical nuclear localization of Smad3. We also show that sequestering of Smad3 within the nucleus prevents its signaling transducer capability. Meanwhile, the unique linker region of Smad3 greatly compromises its affinity in association with Smad4 upon signaling activation. Smad2 is absolutely required for proper epiblast and three germ layer differentiation from embryonic stem cells. However, Smad3 is marginally involved in these events mediated by canonical TGFβ/Smad2 signaling. In contrast, Smad3 is important for normal neural specification, an early developmental event supposes to be inhibited by TGFβ related signals. Furthermore, this neural potentiation role of Smad3 depends on its nuclear distribution property which is unrelated to TGFβ activation. Chip-seq and reporter analyses show that both Smad2 and Smad3 bind to the conserved Smads binding element (SBE), but they show nonoverlapped target gene binding specificity and differential transcriptional activity. We thus conclude that Smad3 differs from Smad2 in multiple aspects, including static subcellular localization, signaling relaying capability and role in early embryonic development.

## Results

### Smad2 and Smad3 show differential subcellular localization in the absence of agonist stimulation

**Figure 1:**
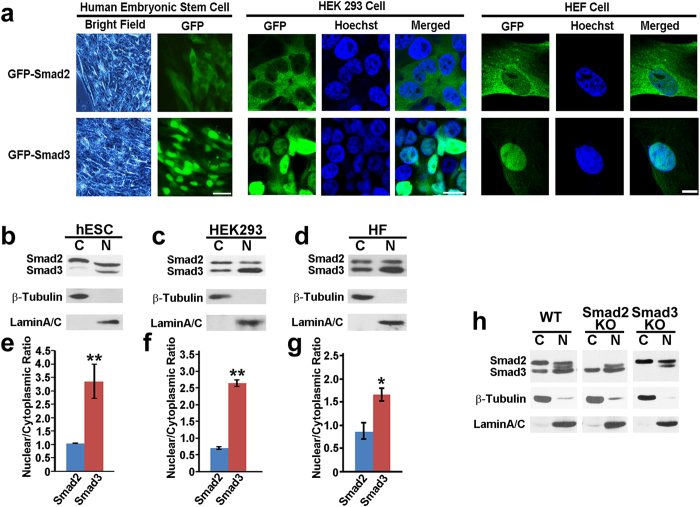
Smad3 is preferentially localized in the nucleus. (**a**) Subcellular localization of GFP-Smad2 and GFP-Smad3 in human embryonic stem cells, HEK 293 cells and human embryonic fibroblast cells. Scale bar, 10 μm. (**b**–**d**) Western blot for cytosolic and nuclear expression level of endogenous Smad2 and Smad3 in hESCs (**b**), HEK 293 cells (**c**), human skin fibroblasts (**d**). β-Tubulin and LaminA/C represent cytosolic and nuclear protein control, respectively. A representative image of three independent experiments is shown. (**e–g**), Quantification data from 3 blots from (**b–d**), respectively. Data are represented as mean +/− SEM. Unpaired two-tailed Student’s t-test. *p < 0.05, **p < 0.01. (H), Western blot for cytosolic and nuclear expression of total Smad2/3 in wild type (WT), Smad2 or Smad3 knockout (KO) HEK 293 cell lines. β-Tubulin and LaminA/C represent cytosolic and nuclear protein control, respectively.

Cytoplasmic to nuclear translocation of R-Smads usually represents activation of TGFβ or BMP signaling. In order to dynamically monitor TGFβ signaling activities during embryonic stem cell (ESCs) maintenance and differentiation, we expressed GFP-tagged Smad2 and Smad3 in human ESCs through lentiviral infection. Consistent with previous studies, GFP-Smad2 was mostly located in the cytoplasm^32^,^33^ (Fig. 1a). However, in surprising contrast to Smad2, a large amount of GFP-Smad3 was distributed in the nucleus. To investigate whether preferential localization of Smad3 in the nucleus is unique to human ESCs, we repeated the experiment in HEK 293 cells and human embryonic fibroblast (HEF) cells. Again, in all cell lines analyzed, GFP-Smad2 showed cytoplasmic distribution, while GFP-Smad3 were more dominantly nuclear localized (Fig. 1a). Endogenous Smad2 and Smad3 also showed distinct subcellular distribution. As shown in Fig. 1b–g, in the absence of TGFβ activation, endogenous Smad2 was mainly detected in the cytoplasm, while a large number of Smad3 was enriched within the nucleus in human ESCs, HEK 293 cells and human skin fibroblast cells. The specificity of the antibody was validated in Smad2 and Smad3 knockout HEK 293 lines generated through CRISPR/Cas9 mediated gene editing (Fig. 1h).

### Biased nuclear enrichment of Smad3 is TGFβ signaling- and Smad4-independent

**Figure 2:**
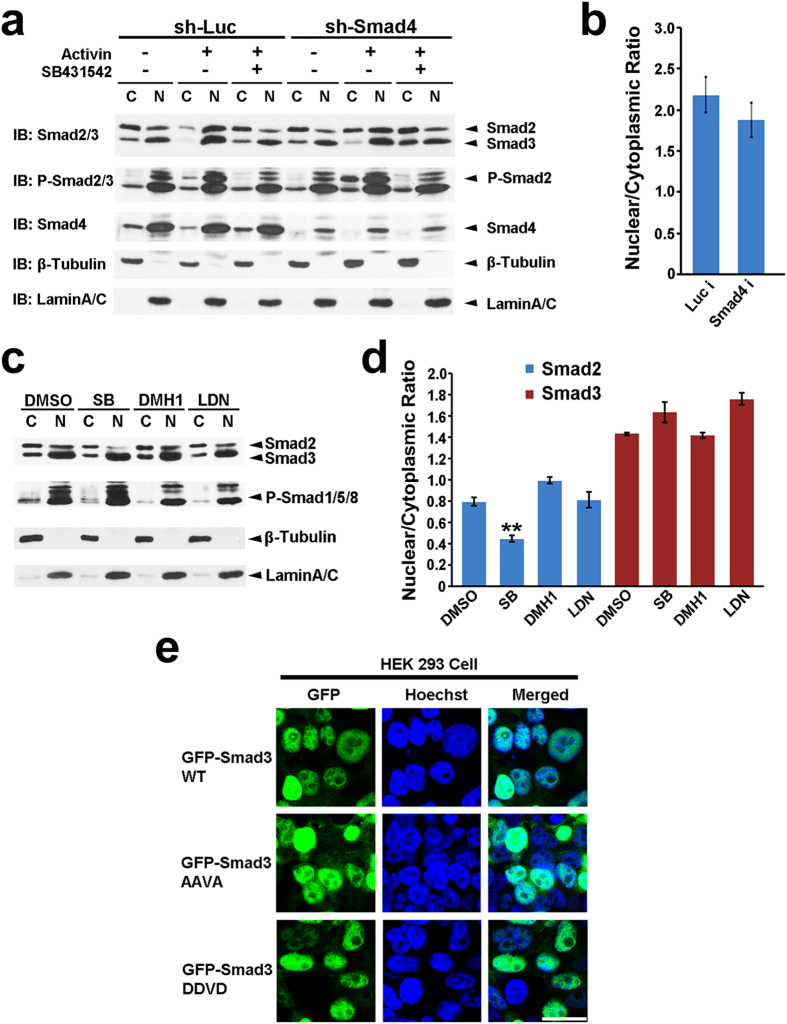
Nuclear localization of Smad3 is TGFβ signaling- and Smad4-independent. (**a**) Western blot for cytosolic and nuclear expression of total Smad2/3, phospho-Smad2 or Smad4 in HEK 293 cells. Cells were infected with Luc or Smad4 RNAi lentivirus to build stable knockdown lines, and were then depleted from serum for 5 hrs, further stimulated with Activin in the presence or absence of SB431542 for 1 hr. A representative image of three independent experiments is shown. (**b**) Quantification data of the nuclear to cytoplasmic ratio of Smad3. 3 blots. Mean +/− SEM. Unpaired two-tailed Student’s t-test. (**c**) Western blot for cytosolic and nuclear expression of total Smad2/3 and phospho-Smad1/5/8 in HEK 293 cells in the presence of SB431542, DMH1 or LDN193189 for 5 hrs. A representative image of three independent experiments is shown. (**d**) Quantification data of the nuclear to cytoplasmic ratio of Smad2 and Smad3 upon SB431542, DMH1 or LDN193189 treatment. 3 blots. Mean +/− SEM. Unpaired two-tailed Student’s t-test. **p < 0.01. (**e**) Confocal images show nuclear accumulation of GFP-Smad3, GFP-Smad3 AAVA mutant (3A) and GFP-Smad3 DDVD mutant (3D). Scale bar, 25 μm.

Smad2 and Smad3 are similarly proposed to act downstream of TGFβ signaling^7^,^11^,^12^,^13^. Upon TGFβ/Activin/Nodal activation, Smad2 and Smad3 C-terminal conserved Ser-Ser-X-Ser motifs (SSXS) are phosphorylated, which promotes their oligomerization with Smad4 and therefore translocates to nucleus for gene regulation. The aforementioned discrepancy of subcellular localization of Smad2 and Smad3 may reflect their differential sensitivity in relaying TGFβ signaling. To test this possibility, HEK 293 cells were depleted from serum for 5 hrs, and further stimulated with Activin for another 1 hr. Upon Activin stimulation, both Smad2 and Smad3 were translocated into the nucleus, suggesting both of them are responsible for TGFβ signals. Knockdown of Smad4 did not affect Smad2 SSXS phosphorylation, whereas it attenuated its nuclear translocation (Fig. 2a). In striking contrast, knockdown of Smad4 could not drive nuclear Smad3 into the cytoplasm (Fig. 2a,b). SB431542 treatment resulted in a great decrease of the nuclear to cytoplasmic ratio of Smad2, suggesting its effectiveness in blocking TGFβ signaling and Smad2 nuclear translocation (Fig. 2a,c,d). In contrast, upon SB431542 treatment, the nuclear to cytoplasmic ratio of Smad3 kept unaffected (Fig. 2a,c,d). These data suggest that the abundant nuclear distribution of Smad3 at the basal level was not originated from its larger capability in relaying TGFβ signals. Treatment of the cells with DMH1 or LDN193189, specific inhibitors for BMPs signaling, could not attenuate nuclear enrichment of Smad3 either, though BMPs may transactivate Smad2/3 in certain cell contexts^34^ (Fig. 2c,d). In addition, mutation of the SSXS motif of Smad3 to AAXA or DDXD to mimic de-phosphorylation or forced phosphorylation state of Smad3 could not change the nuclear localization bias of GFP-Smad3 (Fig. 2e). Collectively, all these results strongly indicate that Smad3 preferentially resides in the nucleus and this distinct nature of Smad3 is independent of TGFβ/BMP signaling, C-terminal SSXS phosphorylation and oligomerization with Smad4.

### Sequestering of Smad3 within the nucleus hindered its role in mediating TGFβ signaling

**Figure 3:**
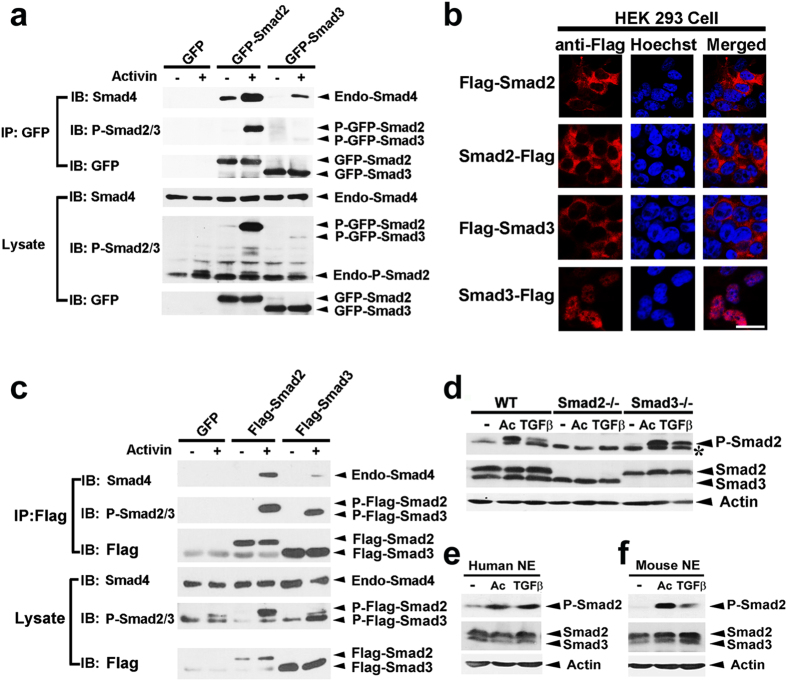
Sequestering of Smad3 in the nucleus hinders its role in mediating TGFβ signals. (**a**) HEK 293 cells stably expressing GFP, GFP-Smad2 or GFP-Smad3 were stimulated with Activin for 1 hr before lysed for GFP immunoprecipitation. The immunocomplexes as well as the lysates were analyzed by Western blotting with anti-Smad4, anti-phospho-Smad2/3 and anti-GFP, respectively. A representative image of three independent experiments is shown. (**b**) Confocal images represent N-terminal Flag tagged Smad2 or Smad3, C-terminal Flag tagged Smad2 or Smad3 subcellular distribution. Scale bar, 25 μm. (**c**) HEK 293 cells were transiently transfected with Flag-Smad2 or Flag-Smad3 and were stimulated with Activin for 1 hr before immunoprecipitation with the M2 beads. The immunocomplexes as well as the lysates were analyzed by Western blotting with anti-Smad4, anti-phospho-Smad2/3 or anti-Flag, respectively. A representative image of three independent experiments is shown. (**d**) Wild type, Smad2 knockout and Smad3 knockout HEK 293 FT cells were stimulated with Activin or TGFβ1 for 1 hr and cell lysates were analyzed through Western blotting. *represents the nonspecific band. (**e,f**) Western blot for phospho-Smad2/3 and Smad2/3 expression in human (**e**) or mouse (**f**) ESCs derived neuroepithelial cells treated with Activin or TGFβ1.

During TGFβ activation, cytoplasmic R-Smads are recruited from the cytoplasm to the membrane-bound activated Type I receptors and therefore transduce extracellular signals. Since a vast majority of Smad3 is distributed in the nucleus even without TGFβ stimulation, we hypothesize that these nuclear localized Smad3 could not sense transmembrane signals. Cells stably expressing GFP, GFP-Smad2 or GFP-Smad3 were stimulated with Activin for 1 hr. After immunoprecipitation with the GFP antibody, phosphorylation of GFP-Smad2 or GFP-Smad3 and their interaction with Smad4 were compared. Upon Activin stimulation, phosphorylation of GFP-Smad2 was significantly increased as compared with the untreated cells (Fig. 3a). Agonist treatment also strongly promoted the interaction of GFP-Smad2 with Smad4 (Fig. 3a). However, under the same conditions, GFP-Smad3 was hardly phosphorylated and its interaction with Smad4 was also minimal (Fig. 3a).

As shown in Fig. 1, GFP-Smad3 located mainly in the nucleus, which was consistent with that of endogenous Smad3. However, when Flag tag was fused to the N-terminus of Smad3, it changed Smad3 distribution from nucleus to cytoplasm (Fig. 3b). Both Flag-Smad2 and Flag-Smad3 now showed similar cytoplasmic distribution, and they also got comparably phosphorylated upon Activin stimulation (Fig. 3c). These data strongly suggest that the cytoplasmic Smad3 could sense TGFβ signals, while, the restricted nuclear distribution of Smad3 significantly abrogates its ability in mediating TGFβ signals. Strikingly, Flag-Smad3 weakly interacted with Smad4 upon agonist stimulation even though it was phosphorylated (Fig. 3c). We also noticed that fusion of Flag tag to the C-terminus of Smad2 or Smad3 did not affect their intracellular distribution characteristics (Fig. 3b). However, C-terminal tag fusion greatly masked Activin-stimulated SSXS phosphorylation and subsequently oligomerization with Smad4 (data not shown).

To verify whether endogenous Smad2 and Smad3 show differential sensitivity in relaying TGFβ related signals, we stimulated HEK 293 cells with Activin or TGFβ1 for 1 hr. As shown in Fig. 3d, either Activin or TGFβ1 treatment triggered robust Smad2 C-terminal phosphorylation. However, endogenous Smad3 was minimally phosphorylated (Fig. 3d). Similar results were obtained in human or mouse ESCs derived neuroepithelial cells (NE) (Fig. 3e,f), supporting our conclusion that Smad3 is less sensitive in relaying TGFβ signals.

### TID domain determines various subcellular distributions of Smad2 and Smad3

**Figure 4:**
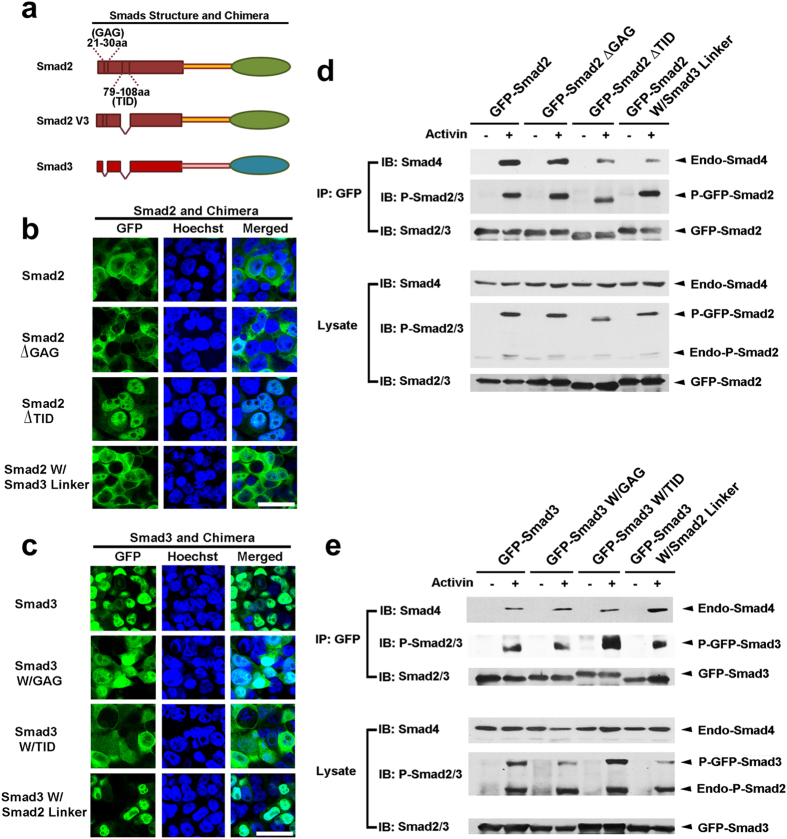
The impaired ability of Smad3 in relaying TGFβ signals results from its distinct MH1 domain and linker region. (**a**) Schematic representation of Smad2, Smad2 V3 and Smad3 protein alignments. (**b,c**) Confocal images of GFP-Smad2, GFP-Smad3 and their chimera mutants. Removal of the TID domain, but not the GAG domain, results in Smad2 nuclear accumulation. Insertion of the TID domain in the MH1 region of Smad3 is sufficient to switch the nuclear localized Smad3 into the cytoplasm. Scale bar, 25 μm. (**d,e**) HEK 293 cells stably expressing GFP-Smad2, GFP-Smad3 or their chimera mutants were stimulated with Activin for 1 hr before immunoprecipitation with the GFP antibody. The immunocomplexes as well as the lysates were analyzed by Western blotting with anti-phospho-Smad2/3, anti-Smad2/3 or anti-Smad4, respectively. Two representative images from three independent experiments are shown.

Smad2 and Smad3 are highly homologous with over 90% amino acid similarity. The main differences between them reside in their linker region and the two additional stretches of amino acids, termed as GAG and TID in the MH1 domain of Smad2^23^ (Fig. 4a). The TID sequence involved in Smad2 is coded by exon3, which is alternatively spliced between full length Smad2 (Smad2) and Smad2 transcript variant 3 (Smad2 V3)^23^,^24^. To test which structural differences between Smad2 and Smad3 contributes to their distinct subcellular distribution, we individually switched the GAG, TID or linker region within Smad2 and Smad3, and their subcellular localization were observed under microscope. As shown in Fig. 4b,c, removal of TID from Smad2 led it accumulated in the nucleus similar to that of Smad3, while insertion of TID to the analogous site in the MH1 domain of Smad3 made it act more like Smad2 and the mutant Smad3 was now mostly enriched in the cytoplasm. Deletion of GAG from Smad2 or insertion of GAG into Smad3 did not affect their normal subcellular localization, nor did the interchanging of the linker regions between Smad2 and Smad3. Thus, the extra TID in Smad2 is responsible for the differential nuclear/cytoplasmic distribution of Smad2 and Smad3.

### The linker region of Smad3 interferes with agonist-stimulated R-Smads/Smad4 oligomerization

As we concluded in Fig. 3, cytoplasmic localization of R-Smads was a prerequisite for sensing transmembrane signals. Removal of the GAG domain in Smad2 or insert GAG domain in Smad3 did not affect their subcellular distribution and these mutants preserved their tendency as of SSXS phosphorylation and subsequent Smad4 binding (Fig. 4d,e). Smad2 with TID domain deletion is mostly localized in the nucleus and it showed compromised phosphorylation and Smad4 binding upon Activin challenging (Fig. 4d). Smad3 with TID domain insertion showed preferential cytoplasmic distribution and increased SSXS phosphorylation as compared with the wild type counterpart (Fig. 4e). However, this mutant did not show increased Smad4 binding tendency in correlation with its higher phosphorylation level, which is very much similar to the Flag-Smad3 construct (Figs 3c and 4e). These data suggest that in comparison to Smad2, Smad3 exhibits a much lower affinity in interacting with Smad4 even under the circumstance when its SSXS motif is similarly phosphorylated. Smad2 W/Smad3 linker was a cytoplasmic protein and it got normally phosphorylated in the SSXS motif after Activin stimulation. However, this chimera demonstrated a much weaker ability in binding to Smad4 (Fig. 4d). On the other side, Smad3 W/Smad2 linker was still mostly nuclear localized and it preserved its weak ability in sensing membrane signals as showed by minimal Activin-stimulated SSXS phosphorylation. Notably, this chimera exhibited a substantially increased ability in interacting with Smad4 during Activin activation (Fig. 4e). All these data suggest that the linker region of Smad3 confers it a weak binding partner for Smad4 and Smad3 is thus a weak downstream effector in relaying TGFβ related signaling.

### Smad3 differs from Smad2 in regulating epiblast development

**Figure 5:**
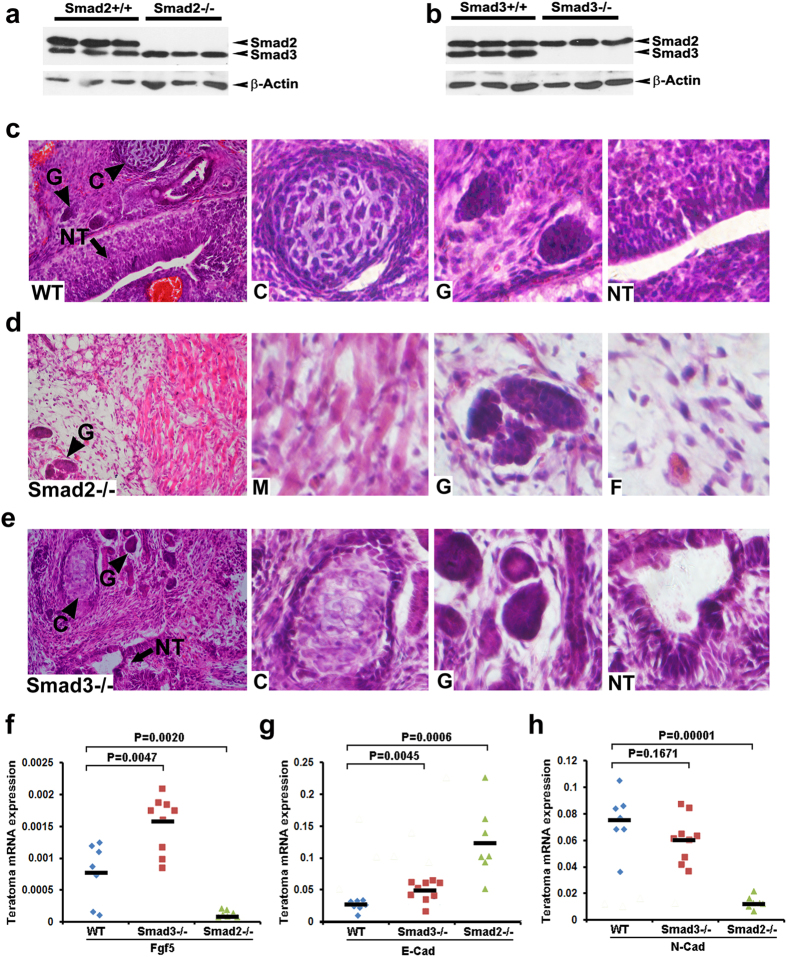
Smad2, but not Smad3, is required for TGFβ signaling regulated epiblast development. (**a,b**) Western blots for expression of total Smad2/3 from WT, Smad2^−/−^ or Smad3^−/−^ mouse ESCs derivatives. (**c–e**) Representative H&E staining images of teratomas generated from WT, Smad2^−/−^ and Smad3^−/−^ mouse ESCs. High magnification images are presented at the right three panels. C, cartilage; G, glomeruli; NT, neurotube; M, muscle; F, fibroblast. (**f–h**) Quantitative RT-PCR data show loss of FGF5, N-Cadherin, while upregulated E-Cadherin expression in Smad2^−/−^ teratomas. WT, 7 samples; Smad2^−/−^, 7 samples; Smad3^−/−^, 9 Samples. Horizontal line, mean. Unpaired two-tailed Student’s t-test.

The well-defined function of TGFβ signaling is more evident in cell fate specification events during early embryonic development. It is widely considered that activation of TGFβ promotes mesendoderm differentiation, while inhibition of TGFβ promotes neural differentiation^35^,^36^. Knockout of Smad2 is embryonic lethal, and mice with Smad3 knockout seem to be phenotypically normal, but recent studies start to uncover some neural abnormalities in these mice^37^. Therefore, a fundamental question is whether Smad2 and Smad3 play equal roles in mediating TGFβ signaling and early lineage specification. We then derived WT, Smad2^−/−^ or Smad3^−/−^ mouse ESCs through heterozygous intercrossing^20^,^38^. The knockout efficiency of Smad2 or Smad3 was verified by Western blotting (Fig. 5a,b).

Teratoma formation analysis is one of the *in vivo* models for studying lineage specification. WT, Smad2^−/−^ and Smad3^−/−^ ESCs were subcutaneously injected into NOD/SCID mice, respectively, and teratomas were harvested 2–3 weeks post injection. H&E staining showed that WT and Smad3^−/−^ teratomas comprised all three germ layers, including cartilage like mesoderm tissues, glomeruli like endoderm tissues and neural tube like ectoderm tissues (Fig. 5c,e). In striking contrast to WT and Smad3^−/−^ teratomas, Smad2^−/−^ teratomas mainly had fibroblasts like tissues and within which scattered with few muscle tissues and endoderm tissues, while neural tube like ectoderm tissues were hardly presented (Fig. 5d). RT-PCR experiments further verified that Smad2^−/−^ teratomas almost completely lack of FGF5 expression, the most important marker gene for epiblast development (Fig. 5f). A bulk of evidence has demonstrated that TGFβ signaling is absolutely required for epiblast development *in vivo* and *in vitro*^39^,^40^,^41^. Therefore, Smad2 is indispensable for normal TGFβ signal transduction and TGFβ/Smad2 pathway triggered primitive ectoderm (epiblast) priming is a prerequisite for proper three germ layer development. The fact that lesion of Smad3 increased rather than decreased FGF5 expression also strengthened the point that Smad3 is not a key effector downstream of TGFβ related signals. RT-PCR analysis also showed that knockout of Smad2 greatly elevated E-Cadherin, while block N-Cadherin expression (Fig. 5g,h). The Switch from E-Cadherin to N-Cadherin has been recognized as a pivotal hallmark of epithelial to mesenchymal transition (EMT), which can also be triggered by TGFβ activation. These results imply that Smad2 is the main mediator of TGFβ signaling in mediating EMT. They also indicate that Smad3 is less involved in this TGFβ induced critical developmental event since knockout of Smad3 had mild effects on E-Cadherin and N-Cadherin expressions as compared with Smad2 knockout groups.

### Smad2 and Smad3 inversely regulate neural lineage specification

**Figure 6:**
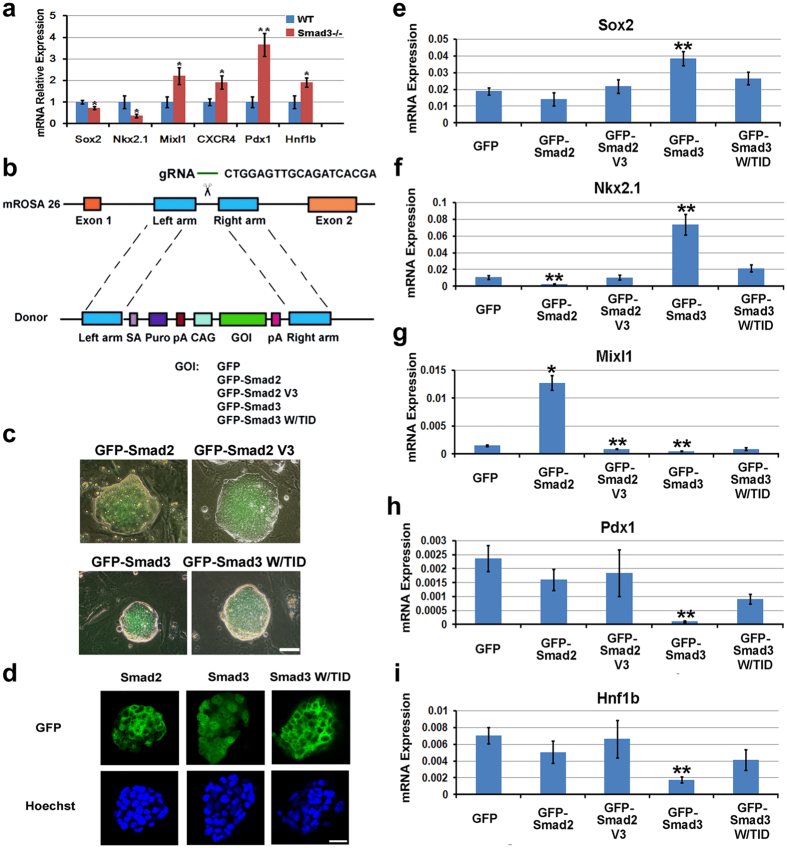
Nuclear localized Smad3 contributes to *in vivo* neural specification. (**a**) Quantitative RT-PCR data show lower neural lineage gene expression while higher mesendoderm gene expression in Smad3^−/−^ teratomas. Quantification data are represented as mean +/− SEM. Unpaired two-tailed Student’s t-test. *p < 0.05, **p < 0.01. (**b**) Schematic strategy for Rosa26 locus knockin. SA, splicing acceptor; Puro, puromycin selection sequence; pA, poly A signal; CAG, cytomegalovirus (CMV) enhancer fused to the chicken β-actin promoter. (**c**) Representative images of expression efficiency and subcellular localization of GFP-Smad2, GFP-Smad2 V3, GFP-Smad3 and GFP-Smad3 W/TID in Smad3^−/−^ mouse ESCs. Scale bar, 50 μm. (**d**) Confocal images of GFP-Smad2 and GFP-Smad3 W/TID show cytoplasmic distribution pattern, while GFP-Smad3 shows nuclear expression pattern. Hoechst stains for nucleus. Scale bar, 25 μm. (**e–i**) Lineage gene expression in teratomas obtained from GFP, GFP-Smad2, GFP-Smad2 V3, GFP-Smad3 and GFP-Smad3 W/TID expressed Smad3^−/−^ mouse ESCs. Quantification data are represented as mean +/− SEM. Unpaired two-tailed Student’s t-test. *p < 0.05, **p < 0.01.

Although the histological studies showed that in Smad3^−/−^ teratomas, all three germ layers were presented, neural tube like structures (rosettes) were lesser than the WT teratomas (Fig. 5c,e). This was confirmed by the mRNA analysis that neural genes, Sox2 and Nkx2.1 were expressed at a lower level in Smad3 knockout teratomas, whereas mesendoderm genes, Mixl1, CXCR4, Pdx1 and Hnf1b were expressed at a higher level (Fig. 6a). These suggest that although knockout of Smad3 does not affect epiblast development or EMT, neural specification is hindered and the differentiated cells are now more biased to a mesendoderm fate.

To confirm that the neural fate promoting effect of Smad3 results from the biased nuclear localization nature rather than TGFβ triggered signaling pathway, rescue experiments were performed by reintroducing GFP, GFP-Smad2, GFP-Smad2 V3 (with TID deletion), GFP-Smad3 or GFP-Smad3 W/TID into Smad3^−/−^ ESCs through Rosa26 locus knockin (Fig. 6b–d). *In vivo* teratoma experiments were also selected to analyze lineage differentiation. GFP-Smad2 overexpression caused lower expression of neural gene Nkx2.1 and higher expression of mesodermal gene Mixl1, supporting the conclusion that TGFβ/Smad2 pathway represses neural but promoting mesodermal development (Fig. 6f,g). GFP-Smad3 rescued all phenotypes caused by Smad3 knockout, that is GFP-Smad3 increased neural while decreased mesendodermal specification (Fig. 6e–i). GFP-Smad3 W/TID was mainly located in the cytoplasm and could not promote neural differentiation, suggesting nuclear distribution is crucial for the neural differentiation promoting effect for Smad3 (Fig. 6e–i). Moreover, GFP-Smad3 W/TID did not behave similarly to GFP-Smad2 in propagating mesendoderm differentiation, mostly likely resulted from its weak ability in oligomerization with Smad4. GFP-Smad2 V3, which does not have the TID sequence and mainly localized in the nucleus similar to Smad3, inhibited Mixl1 expression but no obvious change of neural genes or endoderm genes were observed (Fig. 6e–i). These results implicate that nuclear localized Smad2 V3 may still vary from Smad3 in lineage specification, and the distinct linker region between them may account for this variation. Together, the rescue experiments strongly indicate that Smad3 and TGFβ/Smad2 inversely regulate three germ layer patterning, and the role of Smad3 in these early developmental events may reside in its biased nuclear distribution nature and its insensitivity to bind to Smad4.

### Smad3 potentiates neural specification through binding to conserved SBE

**Figure 7:**
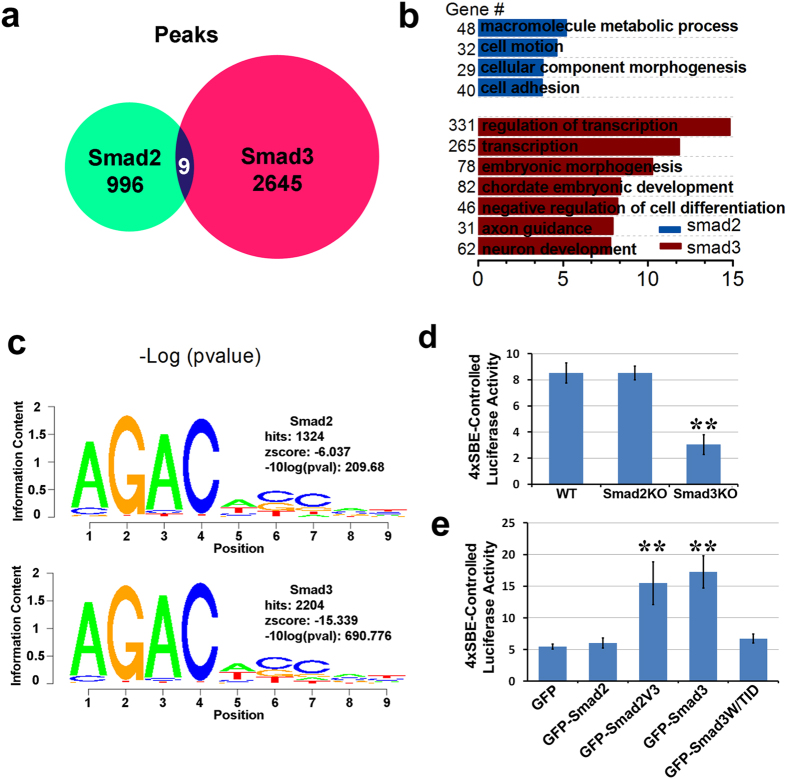
Smad2 and Smad3 share similar DNA binding motifs but show different transcriptional activities. (**a–c**) GFP, GFP-Smad2 and GFP-Smad3 constitutively expressed Smad3^−/−^ mouse ESCs were differentiated to day6 neuroepithelia and collected for Chip-Seq with an anti-GFP antibody. Numbers of aligned peaks are shown in (**a**). GO analysis is shown in (**b**). Top matched de novo motifs surrounding Smad2- and Smad3-bound peaks are show in (**c**), and both Smad2 and Smad3 share similar binding sequence preference. (**d**) Basal 4 × SBE luciferase activity in wild type, Smad2 knockout and Smad3 knockout HEK293 cells. Quantification data are represented as mean +/− SEM. (**e**) Basal 4 x SBE luciferase activity in Smad4 knockout HEK293 cells overexpressed with GFP, GFP-Smad2, GFP-Smad3 or their related mutants. Quantification data are represented as mean +/− SEM. Unpaired two-tailed Student’s t-test. **p < 0.01.

To study the mechanism that how Smad3 facilitate neural specification, we differentiated GFP, GFP-Smad2 and GFP-Smad3 expressing cells with the Smad3^−/−^ background for 6 days toward a neural fate. The cells were then crosslinked and the sonicated lysates were immunoprecipitated with the GFP antibody for further Chip-Seq analysis. We totally found 1005 Smad2 binding peaks and 2654 binding peaks for Smad3 (Fig. 7a). The larger number of Smad3 binding peaks was coinciding with preferential nuclear distribution of Smad3 as compared with Smad2. Interestingly, only 9 among these occupied peaks are shared with both Smad2 and Smad3, suggesting Smad2 and Smad3 may have distinct binding targets. Smad2 enriched genes were largely related to tissue homeostasis, such as cell metabolism, morphogenesis and adhesion (Fig. 7b). While, Smad3 target genes were more related to transcriptional regulators of embryonic development, especially neural development (Fig. 7b). We next performed de novo motif searching in regions surrounding Smad2 and Smad3 binding peaks. The aligned motifs enriched in both Smad2 and Smad3 binding areas mostly have the 5′–AGAC-3′ core sequences, identified as Smad-binding element (SBE) in both *in vitro* and *in vivo* studies for interaction with the MH1 domain of Smads^22^ (Fig. 7c).

Since both Smad2 and Smad3 bind to the conserved SBE, we analyzed their transcriptional activity through luciferase assay. We first analyzed their basal transcriptional activity in HEK293 cells transiently transfected with 4 x SBE-Luc reporter vector and comparison was made in wild type and Smad2 or Smad3 knockout cells. DMH1 was added to the cells in order to minimize the transcriptional activity mediated by BMPs. As shown in Fig. 7d, knockout of Smad3, but not Smad2, showed a significant decrease in the luciferase activity, suggesting Smad3 may be transcriptionally active even in the absence of agonist stimulation. To fully prove the signaling unrelated transcriptional activity of Smad3, we overexpressed GFP-Smad2, GFP-Smad3 or TID chimeras in Smad4 knockout HEK293 cells (Fig. 7e). Indeed, GFP-Smad3, but not GFP-Smad2 elevated the luciferase activity in the absence of TGFβ stimulation. Insertion of TID domain in Smad3 or removal of TID domain in Smad2 switched over their transcriptional activity, indicating the requirement of nuclear distribution of this signaling and Smad4-independent transcriptional activity (Fig. 7e).

## Discussion

In this study, we provide evidence and show that Smad2 and Smad3 have different potentials in relaying TGFβ related signals. Smad2 is a cytoplasmic protein and can be phosphorylated by the activated type I receptor. Phosphorylated Smad2 holds a robust affinity in binding to Smad4 and the Smad2/Smad4 oligomer routinely translocates into the nucleus for target gene regulation. In striking contrast, Smad3 localizes in the nucleus even under the static state and this biased subcellular localization makes it a weak signaling transducer since it cannot easily be recruited to the activated receptor and can hardly get phosphorylated. In addition, Smad3 interacts with Smad4 weakly even when the SSXS motif gets normally phosphorylated. Interchanging of the TID domain or the linker region in between Smad2 and Smad3 switches their subcellular distribution tendency, SSXS phosphorylation capability and properties in oligomerization with Smad4. Furthermore, in a genome-scale CRISPR-Cas9 knockout screening in HEK293 cells, we failed in an attempt to find candidates proteins in helping with static Smad3 nuclear distribution (Supplementary Fig. 1)^42^. All these data reveal that the intrinsic structural differences between Smad2 and Smad3 contribute to these distinct natures.

Functional assays also show clear differences between Smad2 and Smad3. Teratoma formation analysis of Smad2^−/−^ ESCs largely recapitulates embryonic phenotypes of Smad2 null mice, i.e. epiblast formation and three germ layer patterning deficiencies. These defects are closely related to TGFβ hypofunction and support the conclusion that Smad2 is the key mediator of TGFβ pathway^39^,^40^,^41^. Smad3^−/−^ ESCs develop epiblast normally and have all three germ layers, suggesting that Smad3 varies significantly from Smad2 and is not an important TGFβ downstream effector.

Inhibition of BMP and TGFβ signals is the core point of the “default” model for controlling neural lineage specification. Small molecule-based dual Smads inhibition paradigm is now widely used for *in vitro* neural differentiation from human ESCs^35^. Based on this model it should be reasonable to hypothesize that lesion of either Smad2 or Smad3 would have similar effects on potentiating neural differentiation, if they have similar roles in mediating TGFβ signals. While in our current study, we uncovered that Smad3 exerts a neural lineage promoting role during ESCs differentiation, which is opposite to the classical role of TGFβ/Smad2 pathway, further supporting the conclusion that Smad2 and Smad3 vary significantly in relaying extracellular TGFβ signals. The neural promoting effect of Smad3 depends on its nuclear localization, since Smad3 with TID insertion localizes in the cytoplasm and fails to rescue Smad3 knockout phenotypes. Moreover, Chip-seq and luciferase assay showed that Smad3 binds to SBE and is transcriptionally active in a signaling independent manner. Future work is needed to investigate how Smad3 regulates neural lineage development in concert with canonical TGFβ/Smad2 pathway at the transcriptional level.

## Materials and Methods

### DNA Construction

Smad2, Smad3 and Smad4 cDNA or their mutants were constructed into the pLVX-Tet-On (Clontech) or pLenti vector for inducible or constitutive over-expression. The sequence for targeted RNAi was constructed into pLKO vector with PGK-GFP-IRES-BSD in the backbone for blasticidin selection. The target RNAi sequences were: Luciferase, cgtacgcggaatacttcga; Smad4, gtacttcataccatgccga. All constructs were verified by sequencing.

### Antibodies

The antibodies used for immunofluorescence, immunoblotting or immunoprecipitation were purchased from Cell Signaling (Smad2/3, 8685; P-Smad2/3, 8828; P-Smad1/5/8, 9511), Sigma (Flag M2, F3165; β-Actin, A5316; β-Tubulin, T5201), Invitrogen (GFP, A6455), Santa Cruz (Smad4, sc-7966; Lamin A/C, sc-7292; Oct 4, sc-5279) and Jackson Immuno Research for secondary antibodies. Hoechst (Sigma, D9542) was used for counterstaining of nucleus.

### Cell culture and maintenance

HEK 293 cells, human embryonic or skin fibroblasts and MEF cells were cultured in Dulbecco’s Modified Eagle Media (DMEM; Gibco/BRL, Gaithersburg, MD, USA) containing 10% fetal bovine serum (FBS, Gibco/BRL). Human ESCs (H9, passages 18–35) were provided by the WiCell Institute and were cultured on irradiated MEFs as previously described^43^,^44^. Mouse ESCs (D3) were cultured in DMEM with 15% fetal bovine serum supplemented with 10^3 ^U/ml leukemia inhibitory factor (Lif, Milipore, ESG1107).

### Neuroepithelial differentiation from mouse and human ESCs

For mouse ESC neural differentiation, half million cells were suspended in DMEM-F12/neurobasal medium (1:1 DMEM-F12/neurobasal medium, 1X N2 neural supplement, 1X lipid concentrate, 1 mM L-glutamine, 0.1 mM β-mercaptoethanol and 60 μg/ml N-acetyl cysteine). After 4–6 days of culture in suspension, neuroepithelial aggregates were collected for Western blotting. Neural differentiation of hESCs was performed following a published protocol^43^,^44^.

### Lentivirus production and transduction of HEK 293 or ESCs

Lentivirus production was described previously^44^. Briefly, HEK 293 FT cells (Invitrogen) were plated in the 10 cm dish overnight and reached at an 80% of confluence before transfection. For each dish, 10 μg of overexpression or RNAi lentiviral vectors, 7.5 μg of ∆8.9 and 5 μg of VSVG plasmids were cotransfected into HEK 293 FT cells through calcium precipitation method. Fresh medium were supplied 16–20 hrs after transfection. 48 hrs later, medium containing pseudoviruses were collected and concentrated through ultracentrifugation. For transduction of HEK 293 cells, cells were incubated with viral supernatant at 37 °C overnight, and then replaced with new medium. For transduction of human or mouse ESCs, cells were incubated with 100 μl of concentrated virus (10^6^ transducing units/ml) at 37 °C for 30 min. The virus and cell mixture were then transferred to the MEF feeder layer overnight before changing medium on the next day. Forty-eight hours after infection, puromycin, G418 or blasticidin was added to the cells for selecting drug-resistant clones.

### Derivation of Smad2 and Smad3 knockout HEK 293 lines

CRISPR/Cas9 mediated gene editing was performed to knockout endogenous Smad2 or Smad3. The gRNAs designed for Smad2 were: CCAGTTGTGAAGAGACTGCT, CGGAGGAGAGCAGAATGGGC; and gRNAs for Smad3 were: CTGGACGACTACAGCCATTC, CGCAGGCATCGAGCCCCAGA. After double gRNAs transfection together with the Cas9 expression vector, HEK 293 cells were serially diluted and clonalized in 96 well plates. Individual clones were expanded and verified the knockout efficiency through Western blotting.

### Derivation of mouse knockout ES cell lines

C57BL/6 Smad2^−/+^ and Smad3^−/+^ mice were intercrossed. Derivation of mouse ES cell lines were processed by incubation of inner cell mass with standard mESC culture conditions supplemented with 3 mM CHIR99021 and 1 mM PD0325901 (Stemgent, 040004 & 040006), and amplified from 96-well plate and 24-well plate to 6-well plate.

### Homologous recombination based gene overexpression in Smad3^−/−^ mESCs

1 × 10^6^ Smad3^−/−^ ES cells were washed twice with PBS, resuspended in 150 μl of Amaxa electroporation reagents, and mixed with 5 μg Rosa26 gRNA, 5 μg pCas9-GFP (addgene, 44719) and 40 μg individual donor plasmids. After incubating in 0.4 cm cuvette (Bio-Rad) on ice for 5 mins, cells were subjected to electroporation (Bio-Rad, Gene Pulser Xcell Total System) at 320 v, 200 μF and ∞ Ω. The electroporated cells were then cultured on irradiated MEF in DMEM with 15%FBS. Recombinated cells expanded under 5 μg/ml puromycin selection were kept for further biological assay.

### Teratoma formation analysis

The whole animal study was approved by the Institutional Review Board of the Shanghai Tenth People’s Hospital. All animal protocols were approved by the Institutional Animal Care and Use Committee of Tongji University. All mice were maintained in a pathogen-free environment throughout the experiments and every effort was made to minimize suffering. For teratoma assay, 5 × 10^5^ of WT, Smad2^−/−^, Smad3^−/−^ mouse ESCs or Smad3^−/−^ ESCs overexpressed with GFP, GFP-Smad2, GFP-Smad2 V3, GFP-Smad3 or GFP-Smad3 W/TID were injected subcutaneously into NOD/SCID mice. 2–3 weeks post injection, animals were anesthetized with avertin, teratomas were excised, fixed with 4% paraformaldehyde/PBS and sliced at 25 μm for hematoxylin and eosin (H&E) staining. Fresh teratomas were also resolved in Trizol for further gene expression analyses.

### Cell fractionation assay

1 × 10^7^ cells were washed by PBS and resuspended in 250 μl of low osmotic buffer with 0.5% NP40 on ice for 5 mins to break the cell membrane. After spinning at 600 g for 5 mins at 4 °C, the nuclear fraction containing pellet was wash by low osmotic buffer twice, and nuclear proteins were extracted by incubation in 250 μl of high osmotic buffer on ice for 30 mins. The supernatant obtained from the first step of centrifugation was further suffered full spinning and collected as the cytoplasmic portion.

### Co-immunoprecipitation and Western blotting

For the analysis of association of Smad2 or Smad3 with Smad4, cells were lysed in 1 ml of cold RIPA buffer for 2 hrs at 4 °C. The particulate fraction was then removed by full centrifugation. 800 μl of supernatant protein was incubated in the presence of 1 μg primary antibody mixed with 30 μl of 50% slurry Protein-G Sepharose beads (Roche) or incubated with anti-Flag M2 beads (Sigma) at 4 °C overnight. The beads were subsequently washed three times with lysis buffer and were solubilized in 1 × SDS-PAGE loading buffer at 50 °C for 20 mins and separated by SDS-PAGE. To analyze cellular protein concentration during different differentiation days, cells were collected at different time points and lysed in RIPA buffer. Total amount of protein was calculated with BCA kit (Thermo Scientific) and normalized with RIPA buffer to 1–2 μg/μl for SDS-PAGE.

### mRNA extraction and RT-PCR

Total RNA was isolated using the Trizol kit (Invitrogen) and RNA concentration was determined by NanoDrop 2000 c (Thermo Scientific). 1 μg of total RNA from each sample was reverse transcribed into cDNA using SuperScript III (Invitrogen) and subjected to real-time PCR (Bio-Rad, CFX Connect Real-Time System) using the Ssofast EvaGreen kit (Bio-Rad). Primer oligonucleotides used for real-time PCR were as follows (Table 1).

### Chip-Seq and bioinformatics analysis

Neuroectodermal cells were fixed with 1% formaldehyde for 10 mins at room temperature for cross-linking, and then quenched by 0.125 M Glycine for 5 mins. Cells were washed with PBS for several times and digested into single cells by trypsin. Total of 1 × 10^7^ cells were extracted by lysis buffer and chromosome DNA was sonicated. 50 μl of sheared chromatin were under reverse cross-linking and the length were verified abundant at 250 bp, ranging from 100 bp to 600 bp, by electrophoresis. 15 μg of sheared chromatin DNA incubated with 3 μl GFP antibody (Invitrogen, A6455) and 30 μl of ChIP grade protein G magnetic beads (Cell Signaling, 9006) at 4 °C overnight. 2% of sheared chromatin was kept as input. To remove of non-specific chromatin interactions, the beads were washed sequentially with low salt buffer, high salt buffer and LiCl buffer. Immunoprecipitated chromatin DNA was then eluted and purified from magnetic beads followed by deep sequencing.

Chip-Seq reads were aligned to mouse mm9 reference genome using bowtie (version 0.12.7), and only uniquely aligned reads with up to two mismatches were used for the subsequence analysis. Smad2-, Smad3-bound peaks were identified by macs14 (version 1.4.2) with a P-value cutoff of 10–4 by using GFP as control and removal input background. Peak enriched regions within a closest TSS were assigned to the relative gene. SeqPos motif tool of Cistrome was used to find motif enriched in plus/minus 300 base pairs region surrounding Smad2- or Smad3-bound peaks center and motif logos were generated from obtained position weight matrices. Gene ontology (GO) analysis for genes was performed using the tool DAVID 6.7. The raw data were deposited in the NCBI’s Sequence Read Archive (accession number, GSE76557).

### Luciferase assay

HEK293 cells were cotransfected with 4 x SBE-Luc, pRL-TK (Clontech), and other plasmids. 48 hrs after transfection, cells were lysed for 15 min at room temperature (Passive Lysis Buffer, Promega). The lysate was analyzed using the Dual-Luciferase Reporter Assay System (Promega). Results are presented as the ratio.

## Additional Information

**How to cite this article**: Liu, L. *et al.* Smad2 and Smad3 have differential sensitivity in relaying TGFβ signaling and inversely regulate early lineage specification. *Sci. Rep.* **6**, 21602; doi: 10.1038/srep21602 (2016).

## Supplementary Material

Supplementary Information

## Figures and Tables

**Table 1:** Primer oligonucleotides used for real-time PCR.

Gene	Forward Primer	Reverse Primer
Sox2	GCCCTGCAGTACAACTCCAT	TGGAGTGGGAGGAAGAGGTA
Fgf5	AAAGTCAATGGCTCCCACGAA	GGCACTTGCATGGAGTTTTCC
E-Cad	CAGGTCTCCTCATGGCTTTGC	CTTCCGAAAAGAAGGCTGTCC
N-Cad	TCCTGATATATGCCCAAGACAA	TGACCCAGTCTCTCTTCTGC
Nkx2.1	TTGCTTTATGGTCGGACCTGGTGA	AGAAGTCGTCCAGCAGTTTGGTCT
Mixl1	ACGCAGTGCTTTCCAAACC	CCCGCAAGTGGATGTCTGG
CXCR4	CCCGATAGCCTGTGGATGGTGGTGT	TTTTGAACTTGGCCCCGAGGAA
Pdx1	CAAAGCTCACGCGTGGAAAG	TGATGTGTCTCTCGGTCAAG
Hnf1b	AGAGGGAGGTGGTCGATGTC	AGCTGATCCTGACTGCTTTTG
