# Supplementary material for: Smad2 and Smad3 have differential sensitivity in relaying TGFβ signaling and inversely regulate early lineage specification
Source: Sci Rep. 2016 Feb 24;6:21602. doi: 10.1038/srep21602 (PMC4764856; doi:10.1038/srep21602)
Supplement: Supplementary Information [file srep21602-s1.pdf]

# Smad2 and Smad3 have differential sensitivity in relaying TGFβ signaling and inversely regulate early lineage specification

Ling Liu, Xu Liu, Xudong Ren, Yue Tian, Zhenyu Chen, Xiangjie Xu, Yanhua Du, Cizhong Jiang, Yujiang Fang, Zhongliang Liu, Beibei Fan, Quanbin Zhang, Guohua Jin, Xiao Yang and Xiaoqing Zhang*

* Corresponding Author,

Tongji University School of Medicine,

E-mail: xqzhang@tongji.edu.cn.

Room 702, 1239 Siping Road, Shanghai 200092, China.

Tel: 86-21-65985003;

Fax: 86-21-65985003.

Running title: Smad3 varies from TGFβ/Smad2

**Supplementary Figure 1.** Genome-scale CRISPR-Cas9 knockout screening in HEK293 cells. HEK293 cells were infected with lentivirus expressing of GFP-Smad2 or GFP-Smad3 and Cas9. Monoclones were selected through serial dilution in 96 well plates. Individual clones coexpression of either GFP-Smad2/Cas9 or GFP-Smad3/Cas9 were verified by GFP expression and Cas9 mRNA expression. GFP-Smad2 clones showed almost exclusive cytoplasmic expression and secondary infection of viruses bearing the sgRNA library (around $10^5$, to knockout coding genes and non-coding RNAs) generated several clones which show increased GFP-Smad2 expression or nuclear localization of GFP-Smad2. However, infection of viral sgRNA library could not yield any clone which showed cytoplasmic expression of GFP-Smad3.

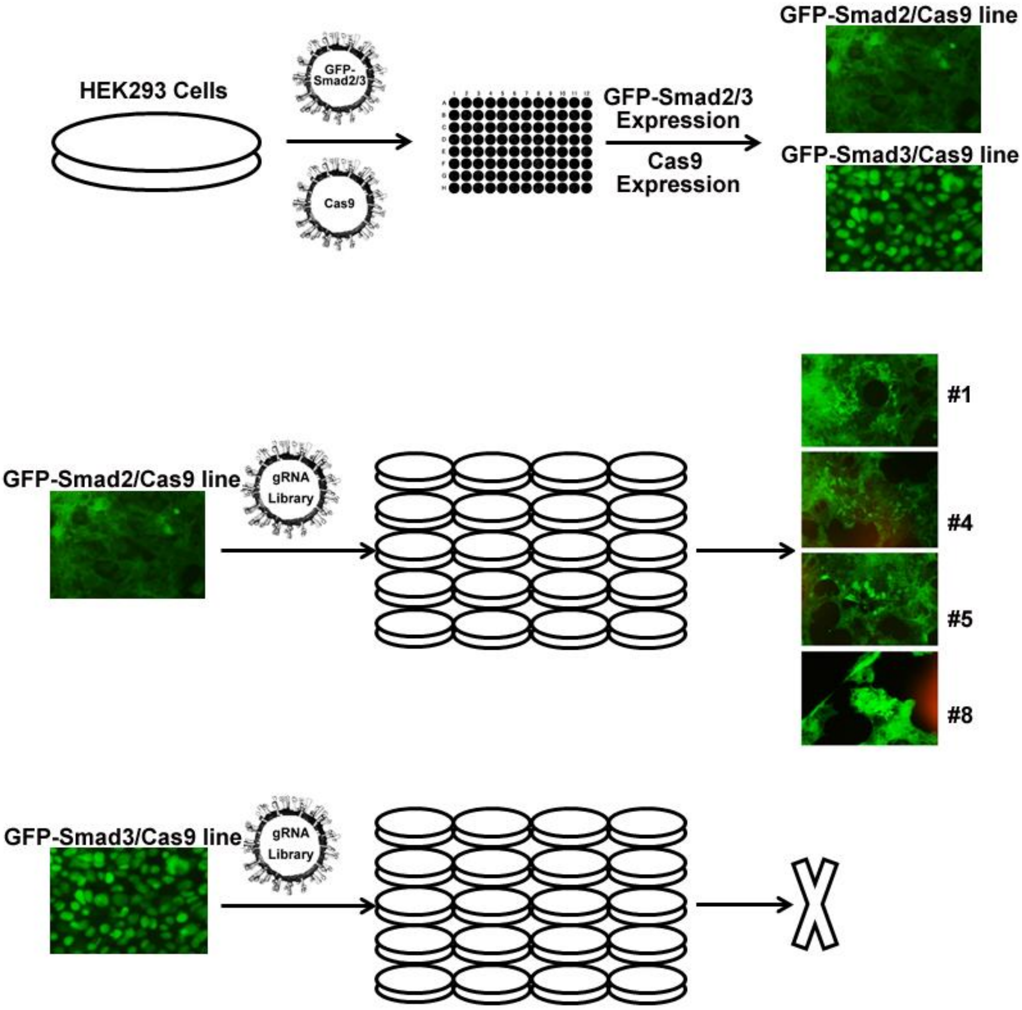
HEK293 Cells
GFP-
Smad2/3
Cas9
GFP-Smad2/3
Expression
Cas9
Expression
GFP-Smad2/Cas9 line
GFP-Smad3/Cas9 line
GFP-Smad2/Cas9 line
gRNA
Library
#1
#4
#5
#8
GFP-Smad3/Cas9 line
gRNA
Library
